# Association between social capital, eHealth literacy, and digital health literacy among undergraduate students in China: a multigroup analysis based on gender differences

**DOI:** 10.3389/fpubh.2026.1839874

**Published:** 2026-05-25

**Authors:** Yangyang Fu, Deyong Lv, Rui Chen, Kaiyuan Feng, Bingsong Li, Qinling Li, Ziwei Qin, Shuyi Yan, Mao Cheng, Zhengna Sun, Yunwei Huang, Jing Wang, Shixue Li, Fanlei Kong

**Affiliations:** 1 Department of Social Medicine and Health Management, School of Public Health, Cheeloo College of Medicine, Shandong University, Jinan, China; 2 NHC Key Lab of Health Economics and Policy Research (Shandong University), Jinan, China; 3 Center for Health Management and Policy Research, Shandong University (Shandong Provincial Key New Think Tank), Jinan, China; 4 Institute of Health and Elderly Care, Shandong University, Jinan, China

**Keywords:** digital health literacy, eHealth literacy, gender difference, social capital, undergraduate students

## Abstract

**Purpose:**

The primary objective of this study was to explore gender differences in the relationships among social capital, eHealth literacy (EHL), and digital health literacy (DHL) among undergraduate students in China, and to support the implementation of the Healthy China and Digital China strategies.

**Methods:**

This study collected 1,151 questionnaires from Chinese undergraduate students through snowball sampling via an online platform. DHL was assessed using the Digital Health Literacy Assessment, EHL was assessed using the eHealth Literacy Scale, and social capital was evaluated using the Chinese Simplified Social Capital Scale. SPSS was used to conduct descriptive statistics, chi-square tests, and independent-sample *t*-tests. AMOS was used to perform structural equation modeling (SEM) to examine gender differences in the relationships among social capital, DHL, and EHL.

**Results:**

The mean DHL score among undergraduate students was 36.27 ± 7.321 (males: 36.65 ± 7.958; females: 36.08 ± 6.975). Social capital showed a positive correlation with DHL, with a stronger standardized direct effect among males (0.107) than females (0.047), although the gender difference was not statistically significant. EHL was positively associated with DHL, with a stronger effect among males (0.847) than females (0.806). Social capital was positively associated with EHL, with a greater direct effect in males (0.410) than females (0.230). The SEM fitness indices were similar between males and females.

**Conclusion:**

DHL among undergraduate students was comparatively high, with males scoring slightly higher than females. Social capital was positively associated with DHL, with a stronger effect observed among males. EHL was positively associated with DHL in both genders, again with a stronger effect among males. Social capital was positively associated with EHL among both males and females, with a greater effect observed among males. These findings provide implications for strategies aimed at enhancing DHL among undergraduate students in China.

## Introduction

With the development of information technology, internet use has increased rapidly in China (1). Increasing internet penetration has made the internet an important resource for accessing and using health information (2). University students are often regarded as a vulnerable population because of cognitive immaturity and limited social experience (3). The inability to effectively identify and utilize online content poses significant risks to university students’ wellbeing and daily functioning (4), particularly given that the Internet usage rate among university students in China is approximately 100% (5). As an important subgroup of university students, undergraduate students require strong capacities in data collection, processing, evaluation, and application to reduce vulnerability and optimize health outcomes (6, 7). Therefore, clarifying the status and determinants of digital health literacy (DHL) among undergraduate students and improving their DHL levels are of considerable social importance (8).

DHL refers to the ability to seek, find, understand, evaluate, and apply health information from digital sources to address health problems (9). DHL integrates multiple subskills, including computer literacy, information and media literacy, science literacy, traditional literacy, and health literacy (10). eHealth literacy (EHL) has been described by the World Health Organization’s global strategy as the set of skills required to seek, find, appraise, and evaluate electronic health information (11). Based on these definitions, EHL mainly emphasizes access to electronic health information (12), whereas DHL places greater emphasis on evaluating and applying digital health information (13). Previous research has regarded digital health as an expansion of the eHealth concept and considered EHL a key component of DHL rather than an interchangeable term (14). From a definitional perspective, the channels used for accessing and processing health information in both DHL and EHL have evolved from earlier electronic platforms, such as smartphones and the internet (15), to broader digital technologies, including remote patient monitoring and wearable devices (16, 17). EHL was developed before the emergence of social media and mobile-based healthcare technologies, whereas DHL reflects more recent advances in digital health technologies (18). To date, few studies have explored the relationship between EHL and DHL, and only one study has reported a positive correlation between them (19).

Social capital can be conceptualized as resources derived from social networks, including social support, information, and prestige (20). Social capital, which is related to social outcomes, has been associated with digital media use among adolescents (21). Higher levels of social capital may also play an important role in obtaining and using health-related information among university students (22). Evidence from China has shown that social capital significantly affects residents’ health literacy in digital life (23), whereas research from Korea has suggested that social capital may contribute to differences in digital literacy (24). However, to date, only one study has empirically confirmed a statistically positive correlation between social capital and DHL (25).

Previous studies have also shown a positive association between social capital and EHL (26). Research by Chang et al. showed that higher levels of social capital were associated with higher EHL (27), whereas Feng et al. reported that individual differences related to social capital were important for EHL (28). Research conducted in Korea further suggested that individuals with stronger social connections tended to have higher EHL levels (29). Evidence from the United Kingdom indicated that differences in social capital contributed to differences in EHL (30). Although one study found a positive association between social capital and EHL among older adults in China (31), research among Chinese undergraduate students remains limited.

The theoretical rationale for gender-stratified analyses integrated the Social Determinants of Health (SDOH) model and Social Role Theory. SDOH refers to the complex circumstances in which individuals are born and live that influence their health, and gender is considered an important and fundamental factor affecting health status (32). Social Role Theory suggests that gender influences the utilization of social capital. Female or communal qualities, such as nurturing and compassion, together with masculine or agentic traits, such as independence, dominance, rationality, and confidence, may influence the pathway between social capital and EHL (33, 34). Consequently, gender may moderate the pathway of “social capital → EHL → DHL.” Therefore, it is necessary to use gender as a grouping variable to explore whether differences exist in the associations among social capital, EHL, and DHL.

In summary, some studies have examined the association between social capital and EHL, whereas few studies have explored the relationships between social capital and DHL, as well as EHL and DHL. However, no study has simultaneously tested the associations among social capital, EHL, and DHL, particularly with respect to gender differences. Therefore, the purpose of this study was to clarify gender differences in the associations among social capital, EHL, and DHL among undergraduate students in China.

## Methods

### Study design and participants

A cross-sectional approach was applied in this study, and snowball sampling without stratification was conducted to enroll the participants. A self-assessment questionnaire was administered to undergraduate students in China for data collection via an online platform (Wenjuan.com) through WeChat between 20 September 2022 and 24 October 2022. The inclusion criteria were undergraduate students enrolled in Chinese universities. All questions were mandatory, and participants who did not complete the questionnaire or provided logically inconsistent responses were excluded. Initially, 1,527 participants from 31 provinces in China joined the online survey. After excluding 55 participants with logically incorrect data, 1,472 responses from university students remained (including undergraduate students and graduate students). Finally, 1,151 undergraduate students from 304 universities were included in this study. All respondents expressed their willingness to participate after understanding the background and purpose of the study.

### Measurements

#### Dependent variable: DHL

The simplified Chinese version of Digital Health Literacy Assessment (DHLA) was used to measure DHL. The DHLA was originally developed in traditional Chinese by Peggy Liu in Taiwan, China (35). The DHLA was divided into two dimensions, namely Digital Health Literacy Self-assessment (SRDHL) and Trust Degree of Online Health Information (TDOHI). SRDHL includes questions 1–6, with response options ranging from “very poor” (1) to “very good” (5). TDOHI includes questions 7–10, with response options ranging from “very unconvincing” (1) to “very convincing” (5). The total DHLA score ranged from 10 to 50, with higher scores representing higher DHL. The Cronbach’s *α* of the DHLA was 0.925 in this study, indicating good internal consistency. Nie et al. demonstrated its reliability and validity with excellent internal consistency in mainland China (36).

### Independent variables

#### Social capital

The China Simplified Social Capital Scale (CSSCS), designed by Xu et al., was used to evaluate social capital (37). The CSSCS has been validated and widely used in China (31, 38). The CSSCS contains two subscales: structured social capital and cognitive social capital. The CSSCS was further divided into six distinct dimensions. Structural social capital includes social participation (questions 1, 2, 3, and 4), social support (questions 5, 6, 7, and 8), and social connection (questions 9, 10, and 11). Cognitive social capital includes trust (questions 12, 13, and 14), cohesion (questions 15, 16, 17, 18, and 19), and reciprocity (questions 20, 21, and 22). Since undergraduate students did not have children, child-related questions were removed. The Cronbach’s *α* for the reduced questionnaire was 0.885. The Cronbach’s α coefficients for the six dimensions were 0.778, 0.734, 0.688, 0.687, 0.850, and 0.805, respectively, indicating good internal consistency of the scale after removing item 9 (39). The structural validity of the CSSCS was assessed by evaluating the correlation coefficient of each item within each dimension. All dimensions showed large effect sizes (correlation coefficient > 0.50) (40), indicating the feasibility of using the CSSCS among undergraduate students in this study. The overall CSSCS score ranged from 22 to 110, with higher scores indicating higher levels of social capital.

#### EHL

EHL was assessed by using the eHealth Literacy Scale (eHEALS), which was designed by Norman and Skinner in 2006 (41) and validated by Lee et al. (42). The eHEALS consists of eight questions divided into two dimensions: Online Health Information Seeking (OHIS; questions 1–4 and 6) and Online Health Resource Appraisal (OHRA; questions 5, 7, and 8). The response options ranged from 1 to 5, where 1 indicated “very inconsistent” and 5 indicated “very consistent.” The summed score of the eHEALS ranged from 5 to 40, with higher scores indicating better EHL level. The Cronbach’s *α* of the questionnaire was 0.958 in this study, indicating good internal consistency.

#### General demographics

This study collected basic demographic information, including age, living area, major, average monthly household income and expenditure, and health assessments compared with peers and with the previous year. Average monthly household income was categorized as less than 3,000 RMB, 3,000–5,000 RMB, 5,000–10,000 RMB, and more than 10,000 RMB. Average monthly expenditure was categorized as less than 1,000 RMB, 1,000–2,000 RMB, 2,000–5,000 RMB, and more than 5,000 RMB. Health assessments compared with peers and with the previous year were both divided into five levels: much worse, worse, roughly equal, better, and much better.

### Statistical analysis

Descriptive analysis was applied to summarize the sociodemographic characteristics, social capital, EHL, and DHL of the undergraduate students. ANOVA and *t*-tests were used to evaluate statistical differences in social capital, EHL, and DHL among undergraduate students with different characteristics. A *p* < 0.05 indicated statistical significance. Statistical Package for Social Sciences for Windows, version 27.0 (SPSS, IBM, Armonk, NY, United States) was used to perform all analyses.

The relationships among social capital, EHL, and DHL were explored using structural equation modeling (SEM) among undergraduate students in China. Maximum-likelihood estimation was applied to determine the best-fitting model. The chi-square test, goodness-of-fit index (GFI), adjusted goodness-of-fit index (AGFI), comparative fit index (CFI), normed fit index (NFI), incremental fit index (IFI), and root mean square error of approximation (RMSEA) were used to evaluate model fit, following the methodology of previous research. Given the large sample size of the study, a non-significant chi-square statistic was unlikely to be observed (43). Therefore, the following fit indices were used in this study: GFI, AGFI, NFI, IFI, CFI, and RMSEA. Good model fit was established when the following criteria were met: *p* > 0.05; GFI, AGFI, NFI, IFI, and CFI > 0.90; and RMSEA < 0.08 (44, 45). SEM was conducted using AMOS (version 28.0; IBM, Armonk, NY, United States).

## Results

### Basic characteristics of the participants

Table 1 summarizes the general sociodemographic characteristics of the undergraduate students. Of the 1,151 undergraduate students, 386 (33.54%) were males, and 765 (66.46%) were females. Among female students, 29.02% were younger than 20 years, and 70.98% were aged 20 years and older, with a mean age of 20.28 ± 1.597 years, which was younger than that of male students (20.61 ± 2.604 years). A total of 71.37% of female students were urban residents, which was higher than that of male students (65.80%). Among female students, 56% were non-medical students, which was higher than that among male students (54.92%). More than half of the respondents reported a monthly family income of over 5,000 RMB in both the male and female groups, and approximately 90% had a monthly personal expenditure of less than 2,000 RMB. In addition, approximately 20% of the participants thought their health was better than that of the previous year or their peers, whereas the majority (80%) considered their health unchanged or worse.

**Table 1:** Basic characteristics of the undergraduate students in this study.

Variable	Gender		χ^2^	*P*
	Male n (%)/mean ± SD	Female n (%)/mean ± SD		
Total	386 (33.54%)	765 (66.46%)		
Age (years)				
<20	103 (26.68%)	222 (29.02%)	26.914^a^	0.020
≥20	283 (73.32%)	543 (70.98%)		
Residence				
Urban	254 (65.80%)	546 (71.37%)	3.754^a^	0.053
Rural	132 (34.20%)	219 (28.63%)		
Major				
MS	174 (45.08%)	326 (42.61%)	0.634^a^	0.426
NMS	212 (54.92%)	439 (57.39%)		
Average monthly household income (RMB)				
0–3,000	49 (12.70%)	80 (10.46%)	1.452^a^	0.622
3,000–5,000	88 (22.80%)	183 (23.92%)		
5,000–10,000	146 (37.82%)	301 (39.35%)		
<10,000	103 (26.68%)	201 (26.27%)		
Average monthly expenditure (RMB)				
0–1,000	111 (28.76%)	176 (23.01%)	10.725^a^	0.013
1,000–2,000	224 (58.03%)	513 (67.06%)		
2,000–5,000	41 (10.62%)	67 (8.76%)		
>5,000	10 (2.59%)	9 (1.17%)		
Health compared to the previous year				
Much worse	27 (6.99%)	31 (4.05%)	12.294^a^	0.015
Worse	108 (27.98%)	233 (30.46%)		
Almost same	150 (38.86%)	349 (45.62%)		
Better	81 (20.99%)	123 (16.08%)		
Much better	20 (5.18%)	29 (3.79%)		
Health compared to peers				
Much worse	23 (5.96%)	14 (1.83%)	21.709^a^	<0.001
Worse	84 (21.76%)	154 (20.13%)		
Almost same	184 (47.67%)	439 (57.39%)		
Better	73 (18.91%)	131 (17.12%)		
Much better	22 (5.70%)	27 (3.53%)		

MS, medical students; NMS, non-medical students. ^a^χ^2^. ^b^ANOVA.

### Structural model

#### Digital health literacy, eHealth literacy, and social capital among undergraduate students

Table 2 presents the DHL, EHL, and social capital scores of the undergraduate students included in this study. Regarding DHL, the average DHLA score among undergraduate students was 36.65 ± 7.96 (males: 36.65; females: 36.08). In the two age groups, older undergraduate students showed higher DHL scores. The DHL level of urban undergraduate students was significantly higher than that of rural undergraduate students (36.74 ± 7.40 vs. 35.20 ± 7.04), which may be related to differences in the allocation of educational resources (46). Undergraduate students in the higher family income groups had significantly higher DHL scores than those in the lower family income groups, with students whose monthly family income exceeded 10,000 RMB showing the highest DHL score (37.32 ± 7.06). Undergraduate students who thought their health was much better than last year had a higher DHL level (40.47 ± 7.87). Similarly, those who thought their physical condition was much better than that of their peers had higher DHL scores than those who thought their physical condition was much worse than that of their peers (41.49 ± 8.099). Statistical differences in DHL scores among undergraduate students were found for residence, major, average monthly household income, health compared to last year, and health compared to peers. The mean EHL score was 28.79 in males and 28.03 in females, whereas the mean social capital score was 66.39 in males and 65.75 in females, indicating higher DHL, EHL, and social capital scores among male undergraduate students.

**Table 2:** DHL, EHL, and SC of the undergraduate students in China.

Characteristics	DHL		EHL		SC	
	Mean (SD)	*P*	Mean (SD)	*P*	Mean (SD)	*P*
Gender		0.217^a^		0.073^a^		0.391^a^
Male	36.65 (7.958)		28.79 (6.924)		66.39 (13.085)	
Female	36.08 (6.975)		28.03 (6.714)		65.75 (11.335)	
Age		0.187^a^		0.162^a^		0.306^a^
<20	35.83 (7.074)		27.85 (6.513)		66.52 (11.253)	
≥20	36.45 (7.413)		28.46 (6.894)		65.75 (12.211)	
Residence		< 0.001^a^		< 0.001^a^		0.165^a^
Urban	36.74 (7.397)		28.80 (6.842)		65.66 (12.286)	
Rural	35.20 (7.037)		27.13 (6.539)		66.88 (11.126)	
Major		<0.001^a^		<0.001^a^		<0.001^a^
MS	37.40 (7.192)		29.61 (6.498)		67.43 (12.105)	
NMS	35.41 (7.307)		27.27 (6.842)		64.84 (11.712)	
Average monthly household income (RMB)		<0.001^b^		0.007^b^		0.080^b^
0–3,000	34.43 (7.997)		26.65 (7.078)		65.27 (12.953)	
3,000–5,000	35.94 (7.489)		27.96 (7.084)		65.53 (12.076)	
5,000–10,000	36.29 (7.089)		28.36 (6.555)		65.86 (11.697)	
>10,000	37.32 (7.059)		29.16 (6.628)		66.81 (11.770)	
Average monthly spending (RMB)		0.091^b^		0.886^b^		0.012^b^
0–1,000	35.75 (7.835)		28.30 (7.078)		64.62 (12.829)	
1,000–2,000	36.26 (7.034)		28.23 (6.565)		65.76 (11.327)	
2,000–5,000	37.24 (7.089)		28.52 (7.210)		67.10 (13.056)	
>5,000	39.11 (9.899)		28.89 (9.427)		66.47 (14.315)	
Health compared to the previous year		0.025^b^		0.012^b^		<0.001^b^
Much worse	35.07 (9.621)		26.72 (8.770)		61.57 (13.944)	
Worse	35.88 (6.998)		27.81 (6.498)		64.25 (11.361)	
Almost same	36.18 (7.178)		28.21 (6.534)		67.01 (11.116)	
Better	36.49 (7.040)		28.76 (6.788)		67.20 (12.158)	
Much better	40.47 (7.874)		32.31 (7.406)		69.29 (17.287)	
Health compared to peers		<0.001^b^		0.018^b^		<0.001^b^
Much worse	35.51 (9.946)		25.65 (9.074)		60.27 (16.015)	
Worse	35.81 (6.968)		27.50 (6.744)		61.74 (10.930)	
Almost same	36.03 (7.285)		28.20 (6.645)		66.44 (10.952)	
Better	36.45 (6.645)		28.72 (5.991)		68.50 (11.758)	
Much better	41.49 (8.099)		33.41 (7.667)		69.44 (16.877)	

MS, medical students; NMS, non-medical students; EHL, eHealth literacy; DHL, digital health literacy; SC, social capital. ^a^*t*-test. ^b^*ANOVA*.

### The structural equation modeling analysis

#### Measurement invariance across gender

Common method bias (CMB) was evaluated via Harman’s single-factor test (47). The analysis showed that 10 factors with eigenvalue > 1 were extracted, and the first factor explained 30.486% of the total variance, which was below the cut-off value of 40%, suggesting the absence of common method bias in the study data (48).

Table 3 presents the fit indices for eight different models, along with the results concerning measurement invariance across gender. To evaluate the appropriateness of gender for group comparison, a preliminary comparison of fit indices between male and female groups was conducted.

**Table 3:** Model invariance test using multi-group nested analysis.

Model	χ^2^	DF	χ^2^/DF	GFI	AGFI	NFI	IFI	CFI	RMSEA	ΔCFI	ΔRMSEA
M1	181.100	58	3.122	0.970	0.942	0.971	0.980	0.980	0.043	–	–
M2	181.100	58	3.122	0.970	0.942	0.971	0.980	0.980	0.043	0	0
M3	181.100	58	3.122	0.970	0.942	0.971	0.980	0.980	0.043	0	0
M4	191.418	65	2.945	0.968	0.946	0.969	0.979	0.979	0.041	−0.001	−0.002
M5	207.983	68	3.059	0.965	0.944	0.966	0.977	0.977	0.042	−0.002	0.001
M6	220.179	69	3.191	0.963	0.941	0.964	0.975	0.975	0.044	−0.002	0.002
M7	220.916	71	3.111	0.963	0.943	0.964	0.975	0.975	0.043	0	−0.001
M8	258.209	84	3.074	0.956	0.942	0.958	0.971	0.971	0.042	−0.004	−0.001

χ^2^, chi square; DF, degrees of freedom; GFI, goodness of fit index; AGFI, adjusted goodness of fit index; NFI, normed fit index; IFI, incremental fit index; CFI, comparative fitness index; RMSEA, root-mean square error of approximation; △CFI, change of CFI; △RMSEA, change of RMSEA. M1 (*n* = 386) = male students; M2 (*n* = 765) = female students; M3 (*n* = 1,151) = unconstrained; M4 (*n* = 1,151) = measurement weights; M5 (*n* = 1,151) = structural weights; M6 (*n* = 1,151) = structural covariance; M7 (*n* = 1,151) = structural residuals; M8 (*n* = 1,151) = measurement residuals. As presented in Table 3, the fit indices were identical for both male and female groups (GFI = 0.972, AGFI = 0.946, NFI = 0.974, IFI = 0.983, CFI = 0.982, RMSEA = 0.041 for M1 and M2). Since the GFI, NFI, IFI, and CFI exceeded 0.9, whereas the RMSEA values were below 0.08, measurement invariance across the two groups was considered acceptable for further testing in the remaining models.

The assessment of measurement invariance used ΔRMSEA, derived from contrasting the less restricted model with its more constrained counterpart. With a sample size greater than 300, ΔCFI values less than 0.010 and ΔRMSEA values less than 0.015 implied that measurement invariance had been successfully established (49).

Measurement invariance was then evaluated using the ΔRMSEA and ΔCFI among models M3 (unconstrained model), M4 (measurement weights model), M5 (structural weights model), M6 (structural covariance model), M7 (structural residuals model), and M8 (latent means invariance model) (50). In model M3, no coefficients were restricted. Model M4 specified that the indicator loadings for the corresponding constructs were equal across groups. Furthermore, M5 restricted both the indicator loadings and the structural coefficients across groups. Model M6 specified that the indicator loadings, structural coefficients, and covariances of the endogenous variables were invariant across the two groups. In addition, M7 constrained the indicator loadings, structural coefficients, covariance of the endogenous variables, and variance of the exogenous variables to be equal across groups. Model M8 tested whether latent construct means were equal across groups, assuming scalar invariance.

In Table 3, the ΔCFI values were 0 between M1 and M2, M2 and M3, and M6 and M7; 0.001 between M3 and M4; 0.002 between M4 and M5, and between M5 and M6; and 0.004 between M7 and M8. The ΔRMSEA values were 0.001 between M4 and M5, M6 and M7, and M7 and M8; 0 between M1 and M2 and between M2 and M3; and 0.002 between M3 and M4 and between M5 and M6, respectively. Since all ΔCFI values were below 0.010 and all ΔRMSEA values were below 0.015, measurement invariance across all models was confirmed. Therefore, the multi-group restriction models demonstrated measurement invariance and could be applied among groups.

#### Model fit indices

**Figure 1:**
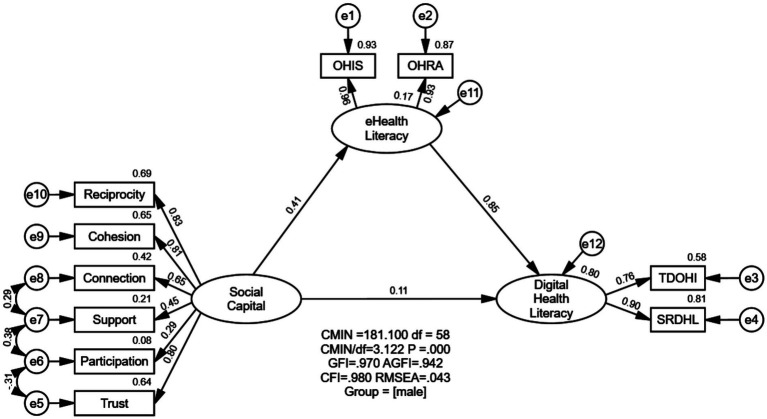
SEM analysis of the association of social capital with DHL and EHL as mediators in male undergraduate students. Note: Rectangles represent observed variables; ellipses represent latent variables; double-headed arrows indicate covariances (or correlations) between variables; single-headed arrows indicate directional path coefficients (pointing from the starting variable to the outcome variable), the numbers on the paths represent standardized path coefficients, and the “e” represents the error terms (measurement residuals) for each variable. SEM, structural equation modeling; CMIN, χ^2^ value; AGFI, adjusted goodness of fit index; DF, degree of freedom; GFI, goodness of fit index; NFI, normed fit index; IFI, incremental fit index; CFI, comparative fit index; RMSEA, root-mean square; SRDHI, self-rated of digital health literacy; TDOHI, trust degree of online health information; OHIS, online health information seeking; OHRA, online health resource appraisal.

**Figure 2:**
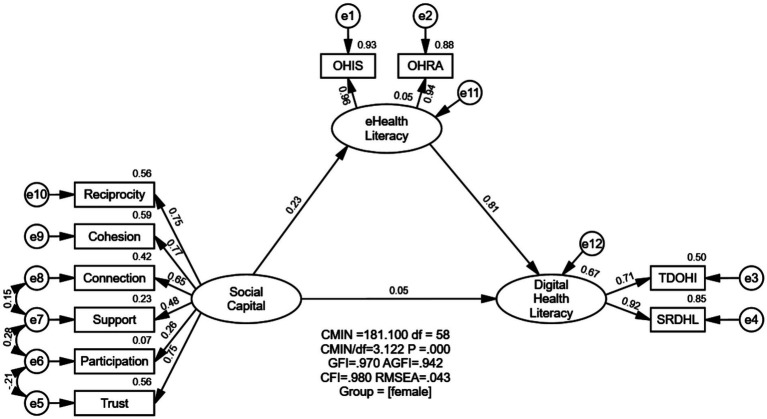
SEM analysis of the association of social capital with DHL and EHL as mediators in female undergraduate students. Note: Rectangles represent observed variables; ellipses represent latent variables; double-headed arrows indicate covariances (or correlations) between variables; single-headed arrows indicate directional path coefficients (pointing from the starting variable to the outcome variable), the numbers on the paths represent standardized path coefficients, and the “e” represents the error terms (measurement residuals) for each variable. SEM, structural equation modeling; CMIN, χ^2^ value; AGFI, adjusted goodness of fit index; DF, degree of freedom; GFI, goodness of fit index; NFI, normed fit index; IFI, incremental fit index; CFI, comparative fit index; RMSEA, root-mean square; SRDHI, self-rated of digital health literacy; TDOHI, trust degree of online health information; OHIS, online health information seeking; OHRA, online health resource appraisal.

Figures 1, 2 illustrate the unconstrained model for male and female groups, respectively. Referring to Figures 1, 2, the model fit estimates were identical for both M1 and M2: GFI = 0.970 > 0.90, AGFI = 0.942 > 0.90, NFI = 0.971, IFI = 0.980, CFI = 0.980 > 0.90, and RMSEA = 0.043 < 0.08, indicating that the model fit the empirical data consistently well for both genders. The chi-square value in AMOS (CMIN) was significant (*p* < 0.001) in this study. However, the CMIN was excluded from the model fit evaluation in this study based of its sensitivity to sample size (51).

#### Relationship between social capital, EHL, and DHL assessed with SEM

Table 4 and Figures 1, 2 support the standardized effects of social capital, EHL, and DHL. In the path model, the total effect comprises both direct and indirect effects. In Figures 1, 2, “social capital → DHL” indicates the direct effect of social capital on DHL, whereas “social capital → EHL → DHL” represents the indirect effect from social capital to DHL through EHL.

**Table 4:** Standardized effects between social capital, eHealth literacy, and digital health literacy by gender.

Variable	Direct		Indirect		Total	
	Female	Male	Female	Male	Female	Male
Social capital → eHealth literacy	0.230^**^	0.410^**^	–	–	0.230^**^	0.410^**^
eHealth literacy → digital health literacy	0.806^**^	0.847^**^	–	–	0.806^**^	0.847^**^
Social capital → digital health literacy	0.047	0.107^**^	0.185^**^	0.347^**^	0.232^**^	0.454^**^

^**^p < 0.01; ^*^p < 0.05.

#### Association between social capital and DHL

As presented in Table 4 and Figures 1, 2 social capital had both direct and indirect effects on DHL. The standardized direct effect from social capital to DHL was observed in both males (0.107, *p* = 0.007) and females (0.047, *p* = 0.097), although no statistically significant association was observed among females. Meanwhile, the indirect effect from social capital to DHL through EHL was positive in both genders, with values of 0.185 (*p* < 0.001) in males and 0.347 (*p* < 0.001) in females. Furthermore, social capital showed a stronger positive effect on DHL among males than females (0.454 for males, *p* < 0.001; 0.232 for females, *p* < 0.001) in this study.

#### Association between EHL and DHL

EHL had a positive direct effect on DHL among both male (standardized direct effect = 0.85, *p* < 0.001) and females (standardized direct effect = 0.81, *p* < 0.001). As mentioned in the Methods section, higher eHEALS scores indicated better EHL. Better EHL status among both female and male undergraduate students was associated with higher DHL. In this study, higher EHL levels were associated with higher DHL among both male and female undergraduate students.

#### Correlation between social capital and EHL

Social capital had a positive indirect effect on EHL for both males and females (0.230 for males, *p* < 0.001; 0.410 for females, *p* < 0.001). This result indicated that higher levels of social capital were associated with better EHL among both male and female undergraduate students in the current study.

## Discussion

### DHL of the participants

The mean value of DHL was 36.27 among undergraduate students, which was similar to previous studies conducted among university students in Iran (52) and Vietnam (53). The moderate level of DHL found in this study was also similar to findings from a Portuguese survey (54) and a study among Korean undergraduate students (55). Regarding gender differences, the mean DHLA score was higher among males (36.65 ± 7.958) than females (36.08 ± 6.975) in this study, which was consistent with previous studies conducted in Germany (56) and Slovenia (57). Such gender differences in perceived digital skill benefits, with males reporting greater positivity, extended to DHL, in which this positivity drove higher DHL levels (58). However, a study conducted among university students in Pakistan found that female students had higher DHL than male students, which differed from the findings of this study (59).

### Social capital and EHL

Social capital had a positive correlation with EHL in this study, which was similar to previous studies conducted in the United States (60) and Israel (61). A study among Chinese nursing students found that social support had a positive relationship with EHL (62), while social support was generally considered to be closely related to social capital (63). In addition, several published studies confirmed that social relationships played a promoting role in improving EHL levels, and may increase low EHL by enhancing social capital (64, 65). Regarding gender difference in the relationship between social capital and EHL, males showed a stronger effect than females in this study. This may be because male university students had higher levels of social capital (66), which supported them in achieving higher EHL. The gender-moderated mechanism accounting for the more pronounced effects in males required further exploration and may arise from differences in social network utilization and disparities in digital accessibility (67, 68).

### Social capital and DHL

Social capital was positively associated with DHL among the Chinese university students in this study, which was similar to findings from a previous study (25) and a study among Portuguese university students (69). Regarding gender difference in the association between social capital and DHL, males showed a stronger effect than females in this study. This may be because males generally had greater social capital than females (70), while higher social capital was associated with increased health information searching (71) and greater health service utilization (72). The male advantage may operate through gender-moderated processes, specifically through the provision of information and support by social capital, which appeared to depend more on the quality of online information than on access itself (73, 74).

### EHL and DHL

Previous studies found that EHL focused more on searching for health information and services (75), whereas DHL extended further by focusing more on health information evaluation and health service utilization (76). A positive and statistically significant path from EHL to DHL was observed in this study, implying that higher EHL levels were associated with increased DHL. EHL provided foundational skills that form the core of DHL, while DHL further developed these skills to enhance information evaluation and health service utilization. Therefore, higher EHL levels were associated with higher DHL levels (77). Regarding gender difference, males showed a stronger effect than females in the relationship between EHL and DHL in this study. This may be because males had higher EHL levels (78) and were better at retrieving health information than females (79). Moreover, better searching skills may indicate a higher likelihood of utilization (80), which may explain the stronger association between EHL and DHL among males.

### Social capital, EHL, and DHL

Previous studies showed that social capital was positively associated with digital health engagement through informational, psychological, and structural factors (81, 82). Specifically, network-based information sharing, interpersonal trust, and skill-sharing provided reliable cues for evaluating online information and overcoming digital anxiety (83, 84). Using SEM, this study hypothesized a sequential pathway in which individuals with higher social capital possessed stronger informational networks and skill-sharing opportunities (85), which fostered EHL, defined as the ability to seek and appraise online health information (86). As EHL improved, individuals were better able to utilize advanced digital health tools for health management (87), ultimately enhancing comprehensive DHL (88).

### Implications

Based on the findings above, several recommendations were proposed to improve DHL among Chinese undergraduate students. First, the positive effect of social capital on DHL indicates the need to build a diversified social support network, including family members, friends, and peers, particularly for female students, to improve their DHL. Second, the positive relationship between EHL and DHL underscores the importance of maintaining and improving EHL among undergraduate students, especially in health information searching skills. Third, social capital and EHL showed a positive correlation. Therefore, EHL among undergraduate students, particularly female students, could be enhanced through gender-tailored digital skills education and health knowledge dissemination via platforms such as women-focused health applications. Enhancing DHL among undergraduate students is essential for improving their health conditions. In terms of health education and national health strategies, these findings suggest that interventions should adopt gender-specific approaches to address the distinct needs of undergraduate students. The observed associations among social capital, EHL, and DHL provide valuable insights for improving DHL among undergraduate students.

### Limitations

Four limitations of this study are listed below. First, the data were obtained via a cross-sectional survey, which cannot be used to determine causal association among the indicators. Second, the utilization of snowball sampling could introduce possible bias originating from the absence of randomization of participants and the potential overrepresentation of certain subpopulations (such as males). Future studies should consider using stratified sampling to achieve better subgroup representation. Third, the ninth item of the CSSCS was removed. Despite the strong reliability of the scale, the validity of the initial scoring procedure for calculating the total CSSCS score should be further explored. Finally, this study used a self-evaluation instrument, which might have caused potential bias. Furthermore, the underrepresentation of students with limited internet access should also be considered.

## Conclusion

DHL was found to be at a relatively moderate level among the undergraduate students in this study. Social capital was found to be positively correlated with EHL and DHL, while EHL was positively associated with DHL among Chinese undergraduate students. These findings may provide empirical evidence for future interventions aimed at enhancing DHL among undergraduate students.

## Data Availability

The raw data supporting the conclusions of this article will be made available by the authors, without undue reservation.
